# Bibliographical

**Published:** 1871-04

**Authors:** 


### BIBLIOGRAPHICAL.

Modern Therapeutics, a compendium of recent formulæ and specific therapeutical directions: By Geo. H. Napheys, A. M. M. D. Second edition, revised and improved. Philadelphia: S. W. Butler, M. D., 115 South Seventh Street, 1871.

We are indebted to the publisher for a copy of the above work. The progress of medicine has been rapid. Many new remedies have been introduced. The work before us shows at a glance this advancement. It contains the most elegant formulas of the most celebrated men of the profession. The practitioner will find it invaluable in more respects than one. The author has not only furnished elegant formulas adapted to the treatment of almost every disease, but he has classified and given specific therapeutical directions in every instance. It is a work on practice—abridged. We recommend the work to the profession. Old and young alike will be benefitted by having it on hand as a book of reference. Price $2.50.

We are also indebted to Dr. S. W. Butler for a copy of *The Physician's Daily Pocket Record*, comprising a visiting list, and many useful memoranda, tables, etc. Dr. Butler, it will be remembered, is the editor of that splendid weekly, the Medical and Surgical Reporter. The Daily Pocket Record is one of the best published. It contains a great variety of useful information. The list of New Remedies alone is worth the price—$1.50.

We also return our thanks to Dr. Butler for a copy of his book of *blank* prescriptions. This is an excellent idea, especially for office practice. Each prescription, by the arrangement, is duplicated, and headings for diagnosis, symptoms, treatment, etc. given. Price $1.25.

*Braithwaite's Retrospect of Practical Medicine and Surgery*: Part LXII—January: This unsurpassed Retrospect holds such a deserved position in the estimation of the profession, and is so favorably known to every medical reader, that to announce its name is to commend it. We do not hesitate to say, that no professional man can afford to be without it. It is worth many times over the price of subscription. Published by W. A. Townsend and Adams. New York: $2.50 a year in advance—postage prepaid. Half-Yearly parts—$1.50.

We have been favored by the publishers, John P. Morton & Co., Louisville, Ky., with a copy of that sterling journal, the American Practitioner, with bound copies for the year 1870. The learning and ability of its editors, Yandell and Parvin, are well known. The volumes before us contain the essence of medical literature for the period it covers. As books of reference to the practitioner, they are invaluable, embracing original and selected articles upon almost every topic connected with medicine and surgery. The original articles are generally excellent—many of them from the pens of Flint, Sr., Parvin, Yandell, Holland, Wible, Whitaker, and others, gentlemen of solid reputation and scientific attainments.

In typographical appearance and binding, these volumes are beautiful specimens of art. We trust that the publishers may become extensively known to Southern medical authors and physicians. They are certainly worthy of their patronage and support.

